# Cytokine response to selected MTB antigens in Ghanaian TB patients, before and at 2 weeks of anti-TB therapy is characterized by high expression of IFN-γ and Granzyme B and inter- individual variation

**DOI:** 10.1186/1471-2334-14-495

**Published:** 2014-09-10

**Authors:** Gloria Ivy Mensah, Kennedy Kwasi Addo, John Amissah Tetteh, Sandra Sowah, Thomas Loescher, Christof Geldmacher, Dolly Jackson-Sillah

**Affiliations:** Noguchi Memorial Institute for Medical Research, University of Ghana, Legon Accra, Ghana; Centre for International Health, Ludwig Maximillians University, Munich, Germany; Division of Infectious Diseases and Tropical Medicine, Medical Center of the University of Munich (LMU), Munich, Germany; German Centre for Infection Research (DZIF), partner site Munich, Munich, Germany

**Keywords:** TB, Biomarkers, Luminex assay, Cytokine profile, ESAT-6/CFP-10 fusion protein, DosR, Week 2

## Abstract

**Background:**

There has been a long held belief that patients with drug-susceptible TB are non-infectious after two weeks of therapy. Recent microbiological and epidemiological evidence has challenged this dogma, however, the nature of the Mtb-specific cellular immune response during this period has not been adequately investigated. This knowledge could be exploited in the development of immunological biomarkers of early treatment response.

**Methods:**

Cellular response to four Mtb infection phase-dependent antigens, ESAT-6/CFP-10 fusion protein and three DosR encoded proteins (Rv1733c, Rv2029c, Rv2628) were evaluated in a Ghanaian TB cohort (n=20) before and after 2 weeks of anti TB therapy. After 6-days *in vitro* stimulation, Peripheral blood mononuclear cell (PBMC) culture supernatant was harvested and the concentration of IFN-γ, Granzyme B, IL-10, IL-17, sIL2Rα and TNF-α were determined in a 6-plex Luminex assay. Frequencies of IFN-γ + CD4 and CD8 T cells were also determined in an intracellular cytokine assay.

**Results:**

All antigens induced higher levels of IFN-γ, followed by Granzyme B, TNF-α and IL-17 and low levels of IL-10 and sIL-2R-α in PBMC before treatment and after 2 weeks of treatment. Median cytokine levels of IFN-γ, Granzyme B, IL-17 and sIL-2R-α increased during week two, but it was significant for only Rv1733-specific production of Granzyme B (P = 0. 013). The median frequency of antigen specific IFN-γ + CD4 T cells increased at week two; however, only the increase in the ESAT-6/CFP-10-specific response was significant (P = 0. 0008). In contrast, the median frequency of ESAT-6/CFP-10- specific IFN-γ + CD8 T cell responses declined during week two (P = 0. 0024). Additionally, wide inter-individual variation with three distinct patterns were observed; increase in all cytokine levels, decrease in all cytokine levels and fluctuating cytokine levels after 2 weeks of treatment.

**Conclusion:**

The second week of effective chemotherapy was characterized by a general increase in cytokine response to Mtb-specific antigens suggestive of an improvement in cellular response with therapy. However, the wide inter-individual variation observed would limit the utility of cytokine biomarkers during this period.

**Electronic supplementary material:**

The online version of this article (doi:10.1186/1471-2334-14-495) contains supplementary material, which is available to authorized users.

## Background

Despite the availability since the 1980′s of an inexpensive, efficacious, and moderately well-tolerated therapy, that can cure 90% of cases, tuberculosis (TB) continues to be a major worldwide health problem, with 8.6 million new cases and 1.3 million deaths in 2012 [1]. One of the factors mitigating against a decline in tuberculosis cases is the long duration of treatment (6 months) which makes adherence difficult and also puts pressure on health care systems in developing countries [2]. Clinical, bacteriological and radiological improvement in TB patients is achieved within 2 months upon effective chemotherapy with a multi-drug regimen [3], making “month 2 sputum culture status” the only acceptable biomarker for TB treatment response [4]. However, waiting for 2 months to reach a decision on the treatment outcome could be damaging to a patient as it unduly delays the need for drug sensitivity testing or changes in treatment regimes. During this 2-month period, primary multi-drug resistant organisms will remain untreated and drug-resistant mycobacteria may have time to develop resistance to additional drugs [5].

Additionally, use of classical microbiological methods like “sputum culture status” as a biomarker for TB treatment response has limited usefulness in children and extra-pulmonary cases, where appropriate quality sputum samples are hard to get. Given the shortcomings associated with using sputum as a sample for monitoring TB treatment response, blood has been proposed as a better alternative. Blood is easier to obtain and assessment of immunological parameters in blood can be done within days and could be well adapted for field use. Measurement of Mtb-specific antigen induced responses in peripheral blood has been used often in recent times, however, most studies using this approach have only focused on IFN-γ as a sole marker of response. It has been proposed that sets of markers rather than a single marker may increase the predictive power [2]. Multiple cytokines assessed at week two, a much earlier time point during treatment than the current stipulated “month two”, could be useful in detecting early cellular response to TB treatment. There has been a long held belief that patients with drug-susceptible TB are non-infectious after two weeks of therapy [6, 7] although recent microbiological and epidemiological evidence has challenged this dogma [8, 7, 9–11]. However, the nature of the Mtb-specific immunological response during this period of TB treatment has not been adequately investigated. It has been demonstrated that Acid fast bacilli (AFB) counts fall by about 20-fold in the first 2 days and by a further 200-fold in the next 12 days to reduce the counts of an initially smear-positive patient to about 10^3^ per ml at 2 weeks of short course chemotherapy [12]. These levels are below the estimates of 10^3.5^ to 10^4^ per ml which are the limits indicating a change from smear-positive to smear-negative, culture-positive in untreated patients [7]. If and whether this is characterized by an improvement in cellular response can potentially be exploited as a possible tool to predict early treatment response. To address this knowledge gap, we identified, in addition to IFN-γ, 5 key cytokines; Granzyme B (grzB), TNF-α, IL-17, IL-10 and sIL-2R-α most of which are important to early control and containment of tuberculosis infection.

Using the Luminex platform, we measured the levels of the 6 selected host markers in the PBMC culture supernatant of TB patients after 6 days long term stimulation with ESAT-6/CFP-10 and 3 DosR antigens (Rv1733c, Rv2029c, Rv2628) known to be immunogenic in African populations [13] before and after 2 weeks of treatment. A profile consisting of high levels (pg/ml) of IFN-γ followed by Granzyme B and TNF-α and low levels of IL-17, IL-10 and sIL-2R-α in PBMC before treatment and at 2 weeks of treatment was observed. We show that there is general improvement in cellular response at 2 weeks of therapy but there is wide inter-individual variation, suggesting that factors other than anti TB therapy account for cellular response observed. These ought to be investigated to further explore the potential to use cytokine response patterns for monitoring treatment response.

## Methods

### Ethical approval

This study was approved by the Institutional Review Board (IRB) of the Noguchi Memorial Institute for Medical Research (NMIMR), University of Ghana, Legon, Accra. Certified Protocol Number (CPN): 030/10-11). All participants were recruited upon written informed consent.

### Study site and patients

A total of 20 newly diagnosed sputum smear-positive TB patients who were yet to begin therapy, were recruited consecutively from three health facilities in Accra, Ghana during May to June 2011. The mean (±standard deviation [SD] age was 34.05 (±9.25) years, and 80% were male. One (1) HIV-positive participant was excluded from the analysis (Table 1). Patients were required to have at least one of two sputum smears positive for acid -fast bacilli (AFB) by direct microscopy to be eligible to take part in the study. From each participant, up to 25 ml of venous blood was drawn using butterfly needles (BD) into 10 ml heparinized vacutainers (BD). Blood samples were taken at two time points; before treatment (baseline), and at 2 weeks of TB treatment. All samples were sent immediately to the laboratories of the Noguchi Memorial Institute for Medical Research (NMIMR) for the appropriate analysis. Demographic information was collected from each participant using a standardized questionnaire. Management of TB cases followed the national guideline i.e. Two months of intensive phase of Rifampicin (150 mg), Isoniazid (75 mg), Pyrazinamide (400 mg) and Ethambutol Hydrochloride (275 mg) and 4 months of the continuation phase with only Rifampicin and Isoniazid.

**Table 1:** **Participant’s characteristics**

Participant	Age group (years)	Gender	Sputum smear microscopy results			
			Diagnosis^^^		Category	Month 2
01	41-50	M	3+	3+	3+	Neg
02	31-40	M	3+	3+	3+	Neg
03	51-50	M	2+	2+	2+	Neg
04	21-30	F	1+	Neg	1+	Neg
05	31-30	M	2+	2+	2+	Neg
06	21-30	M	3+	SC	3+	SC
07*	31-40	M	1+	1+	1+	Neg
08	51-60	M	3+	3+	3+	Neg
09	21-30	M	2+	2+	2+	Neg
**10** ^**#**^	21-30	F	2+	2+	2+	Neg
11	31-40	M	3+	3+	3+	Neg
12	21-30	M	1+	1+	1+	Neg
13	31-40	F	3+	3+	3+	Neg
14	41-50	M	3+	3+	3+	Neg
15	31-40	M	3+	3+	3+	Neg
16	31-40	M	SC	SC	SC	Neg
17	31-40	M	SC	SC	SC	Neg
18	31-40	M	3+	3+	3+	Neg
19*	20-30	M	SC	Neg	SC	Neg
20	21-40	M	SC	Neg	SC	Neg
**Average**	**34.05**	**M (80%)**			**+ (100%)**	**(+) 5%**
**Range**	**[21–55]**					

* *M. africanum* (MAF) ^**#**^HIV + participant.

SC (Scanty) < 10 AFB per 100 fields ^^^Showing two smear results required for TB diagnosis.

### *M. tuberculosis*antigens

The recombinant *M. tuberculosis* proteins evaluated in this study were; ESAT-6/CFP-10 fusion protein (a combination of the two most immunodominant proteins of the RD1 region of MTB) and Rv1733 (possible trans membrane protein of 210 aa), Rv2029 (Phospho fructo kinase B *(pfkB*) of 339aa), Rv2628 (Hypothetical protein of 120aa) which are proteins of the Dormancy Survival Regulon (DosR) of Mtb and which have been used previously and are known to be immunogenic in some African populations [13]. These “*stage specific*” *M. tuberculosis* antigens were selected and produced at the Department of Infectious Diseases, Leiden University Medical Center, Leiden, The Netherlands [14]. ESAT-6/CFP-10 was tested on PBMC from all participants whilst Rv1733, Rv2029 and Rv2628 were tested in 18, 11 and 10 participants respectively. In all cases, 1 ug/ml of *Staphylococcus* enterotoxin B (SEB) (Sigma: Cat. No. S4881) was included as a positive control, while growth medium [(RPMI 1640 GIBCO, lot no-3094131), 10% FBS (Fetal Bovine Serum (SIGMA: catalog no. F9665), 1% Penicillin/Streptomycin (P/S)] was used as a negative control. All Mtb antigens were received in a dehydrated form and were reconstituted with sterile growth medium and used at a final concentration of 5 μg/ml. The working concentration of 5 μg/ml was chosen based on a pilot study which showed that while a concentration of 2.5 μg induced minimal secretion of IFN-γ, there was no significant difference between the secretion at 5 μg and 10 μg.

### PBMC isolation

PBMC was isolated from heparinized whole blood following standardized procedures [15]. Briefly, using sterile 10 ml disposable pipettes (Sarstedt), blood from the three 10 ml vacutainers per participant was transferred into a sterile 50 ml centrifuge tube (GBO) labeled with the participant unique identification number. An equal volume of pre-warmed (37°C) RPMI 1640 (GIBCO) was added to the blood in the falcon tube to achieve a 1:1 dilution and mixed gently. The diluted blood was layered gently onto 15 ml of Histopaque (SIGMA Lot No. H8889) without breaching the Histopaque-blood barrier in a ratio of 2:1 for blood and Histopaque. Both the blood and Histopaque were used at room temperature. The tubes were centrifuged at 800 g for 30 min at room temperature with the brake off. The milky-white ring of PBMC between the Histopaque (transparent) and plasma (yellow) was then aspirated with a sterile pastette into sterile 50 ml tubes, and washed twice with pre-warmed Hank’s Balanced Salt Solution (SIGMA Lot No. H9394). The cells were finally suspended in 1 ml of filtered growth medium for counting using 1 in 2 dilution with 0.4% Trypan blue (GIBCO: Cat. No. 15250–061).

### PBMC culture

Culture plates were prepared by adding 250 μl of antigen suspension at 10 ug/ml to appropriate wells on the culture plates (Nunc; catalog no. 152640) in duplicates. After counting, cells were re-suspended in pre-warmed sterile filtered growth medium at 2million cells per ml and aliquots of 250 μl (500,000 cells) added per well of antigen and growth medium (negative control) to achieve a final concentration of 5 μg/ml of antigen in 500 μl volume per well. Blank spaces were filled with HBSS to prevent evaporation. The plate was covered and sealed with Micropore tape and incubated for 6 days at 37°C in a 5% CO^2^ incubator. The culture form was then filled indicating the subjects IDs, start and end date. The positive control antigen (SEB) was added on the 4^th^ day of culture. On the 5th day of culture, the supernatant was collected and stored at -80°C for further analysis and 5 μg/ml of Brefeldin A (Sigma: Cat. No. B7651) was added to the cells for 18–24 hours until the 6th day when they were then pooled into appropriately labeled FACs tubes (BD Falcon Cat No: 352052) for intracellular cytokine staining and flow cytometry.

### Phenotypic analysis by intracellular flow cytometry

Each well was washed out gently with sterile FACS buffer (1 X PBS, 1% HI-FCS, 0.1% NaN_3_) to completely collect all cells. All tubes were placed on ice ensuring cells are kept cold during staining. Two (2mls) of FACs buffer was added per tube and centrifuged for 5 minutes at 1250 rpm. The supernatant was decanted and the pellet re-suspended in 100 μl FACS buffer and incubated at 4°C in the dark for 30 mins wrapped in aluminum foil with the appropriate amount of monoclonal antibody (Mab) for surface staining: 3 μl of anti -CD4-peridinim chlorophyll (PerCP) (BD: Cat No. 345770); and 2 μl of anti- CD8-allophycocyanine (APC) (BD: Cat no. 561421). After incubation, 2 ml of FACs buffer was added per tube and centrifuged and decanted as before. The cell pellet was then re-suspended in 250 μl of BD Cytofix/Cytoperm (Cat. No. 554722) at room temperature in the dark for 15 minutes to fix the cells for intracellular staining. After fixing, cells were permeabilized by washing 2 times with Iml of 1X BD Perm/Wash buffer (Cat. No. 554723). For the second wash, the cells were incubated with the perm wash for 25 minutes prior to centrifugation at 1800 rpm. The supernatant was decanted, the pellet re-suspended in the residual perm wash (approx. 50 μl) and 1 μl of anti- IFN-γ-fluorescein isothiocyanate (FITC) (BD: Cat No. 554551) added to the tubes and incubated for 30 minutes in the dark. After incubation, 2 ml perm wash was added and centrifuged as before and the supernatant decanted. The pellet was then re-suspended in 0.3 ml of FACS flow solution and the cells were acquired immediately on a FACS Calibur (BD). Using FLOWJO software Version 7.6.2 (Tree Star Inc, USA), we defined an R1 gate for lymphocytes in a dot plot of Forward Scatter Chanel (FSC) versus Side Scatter Chanel (SSC). To identify CD4+ and CD8+ T cells, events from R1 were analyzed in a plot of CD4-PerCP vrs CD8-APC (R2). Finally, gated CD4+ and CD8+ T cells were analyzed for IFNγ-FITC. Data were reported as percentages of IFN-γ + CD4 and CD8 T cells. Compensation settings were defined using anti-mouse kappa Comp Beads (BD Biosciences) stained with each fluorochrome–conjugated antibodies.

### Human 6-PLEX (IFN-γ, TNF-α, IL-17, IL-10, sIL-2R-α and Granzyme B) assay

Six-day culture supernatant previously harvested and stored at -80°C were brought to room temperature to thaw slowly until all ice had completely melted. Whilst still ice cold, the samples were centrifuged to remove cell debris after which an amount was dispensed into new tubes and transported on ice for analysis. The multiplexing analysis was performed using the Luminex™ 100 system (Luminex, Austin, TX, USA) by Eve Technologies Corp. (Calgary, Alberta- Canada). The six markers were measured in the cell culture supernatant using an Affymetrix Human Cytokine/Chemokine Custom plex kit (Affymetrix, Inc, Santa Clara, CA, USA) according to the manufacturer’s protocol. The 6-plex consisted of IFN-y, IL-10, IL-17, IL-2Rα, Granzyme B, and TNF-α. The assay sensitivities of the 6-plex markers ranged from 0.1 – 0.4 pg/ml, and 5 pg/ml for IL-2Rα and Granzyme B.

### Determination of positive responses

For each antigen, values of S/U (*where S/U is defined as follows: cytokine concentration in antigen- stimulated cultures divided by the cytokine concentration in un-stimulated (negative control) as described in* [16] that were greater than or equal to 2 were considered positive responses. In experiments where the concentrations of cytokines in control cultures lacking antigens were not detectable, the S/U values were determined by dividing the concentration of a given cytokine in antigen-stimulated cultures with the minimum detectable concentration of the same cytokine.

### Statistical analysis

Data was entered into Microsoft Excel 2007 (Microsoft Corp., USA) for analysis or transported to Graph pad PRISM version 4 (GraphPad prism software Inc., California, USA) for analysis and graphs. The minimum detectable concentrations of the cytokines or soluble mediator in this assay were 5.7, 0.2, 0.26, 3.35, 1.39, 2.13 pg/ml for Granzyme B, IFN-γ, IL-10, IL-17, sIL-2R-α and TNF-α respectively. The Mann–Whitney U test was used to compare the median concentration of each cytokine in pg/ml at baseline and after 2 weeks of TB treatment and to compare the cytokine secretion profile of antigens associated with the active (ESAT-6/CFP-10) and latency (Rv1733) stages of TB infection. The Wilcoxon signed rank text was used to compare cytokine expression at baseline and 2 weeks of treatment for individuals (n = 9) with samples for both time points available (Matched analysis). The Wilcoxon rank sum test was used to compare cytokine responses in single patients at baseline and after 2 weeks of treatment. Uni-variate analysis was done for “age” and “sputum smear result at diagnosis” with cytokine response to antigenic stimulation to determine cause of individual variation in response observed. In all cases P values less than 0. 05% were considered significant.

For flow cytometric analysis, percentage of IFN-γ + cells were calculated by subtracting the percentage of IFN-γ + cells in un-stimulated cultures from stimulated ones. A threshold of 0.2% IFN-γ + CD4/ CD8 T cells defined positive T-cell responses against antigens. Samples with a negative SEB response were excluded from the analysis. Differences in the percentage of IFN-γ + CD4/CD8 T cells to antigenic stimulation were analyzed using the nonparametric Mann–Whitney U-test. P–values of less than 0.05 were regarded as significant.

## Results

### Participants’ characteristics

All twenty (20) participants were sputum smear positive, however, they were categorized based on AFB smear grading as 3+ (one or both smears were 3+), 2+ (one or both smears were 2+ or lower), 1+ (one or both smears were 1+ or lower) and SC (one or both smears was scanty “less than 10 AFB per field” or negative). Accordingly, nine (9) were classified as 3+, four (4) as 2+, three (3) as 1+ and four (4) as SC (scanty). Eighteen (90%) of the participants were infected with MTB and the remaining two were infected with *M. africanum* (MAF). All except one subject converted to a negative sputum smear at 2 months of treatment (Table 1).

### Antigen-induced secretion of cytokines by PBMC in response to “stage specific” mycobacterial antigens

To ascertain the immunogenicity of each antigen in our active pulmonary TB cohort, we compared the concentration of cytokine (pg/ml) induction in the stimulated to that of the un-stimulated culture. The cytokine concentrations were significantly higher (P < 0.05 to P < 0.001) in stimulated than un-stimulated cultures for all antigens tested at both baseline and after two weeks of therapy (data not shown).

### Positive responders and median cytokine concentration

**Figure 1:**
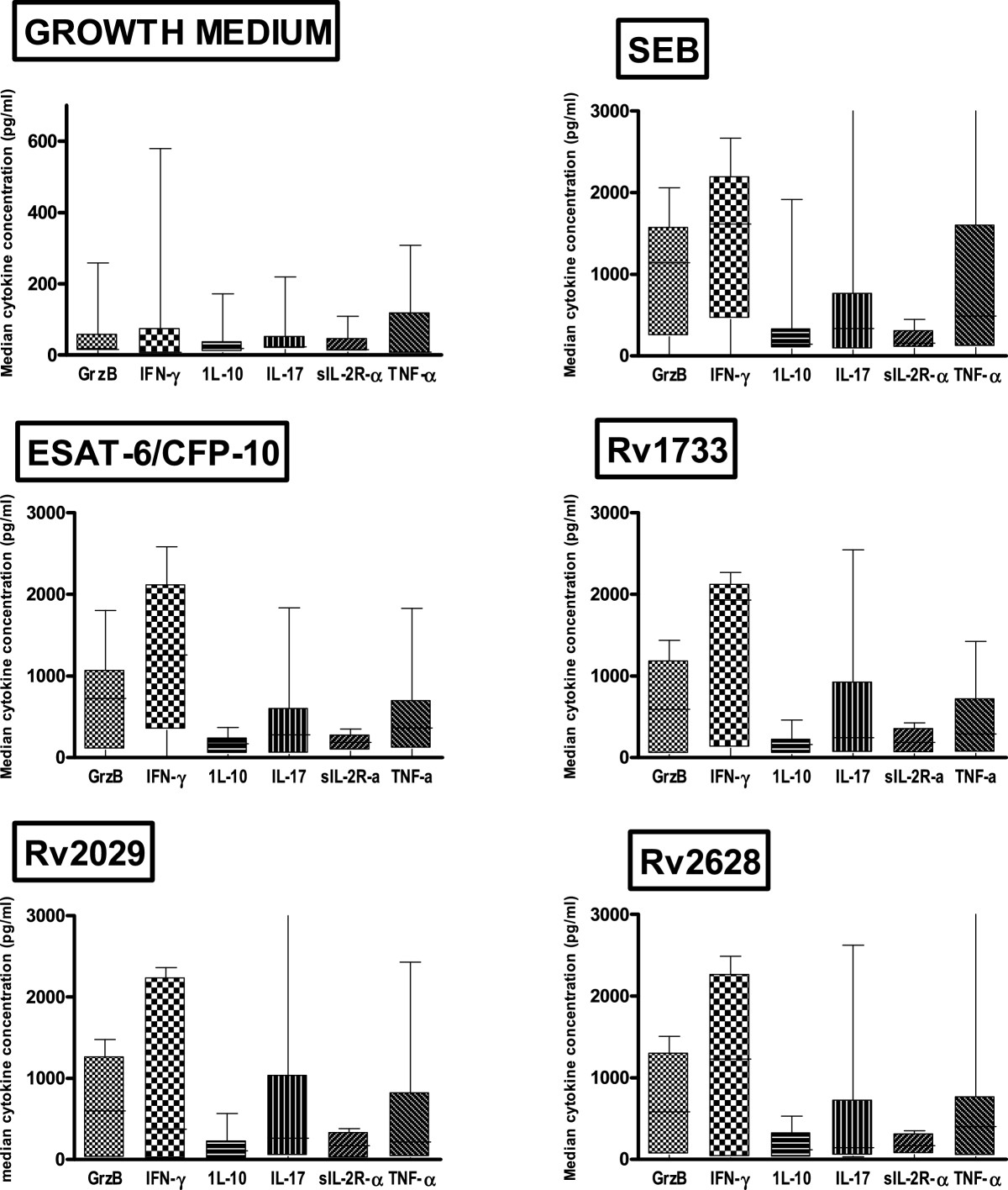
**High level of IFN-γ and Granzyme B are secreted in response to all antigens.** Freshly isolated PBMC obtained from sputum smear positive TB patients (n = 19) at baseline (before treatment) were stimulated *in vitro* with the stage specific *M. tuberculosis* antigens; ESAT-6/CFP-10, Rv1733, Rv2029, Rv2628 (latency associated), positive control *Staphylococcus* enterotoxin B (SEB) and negative control (Growth medium) for 6 days. The harvested supernatant were used in a six-plex Luminex assay for of Granzyme B (GrzB), IFN-γ, IL-10, IL-17, sIL2Rα and TNF-α. The box plots show the 25th, 50th, and 75th percentiles, and the whiskers represent the minimum and maximum levels of cytokine (pg/ml) induced by each stimulus. In response to all antigens, high levels of IFN-γ followed by Granzyme B and TNF-α and low levels of IL-17, sIL2Rα and IL-10 were observed.

Determining the proportion of positive responders to *M. tuberculosis* antigens is critical to prioritizing the antigens as there continues to be a search for the TB antigen with universal immunogenicity. The positive control SEB induced more positive responses than ESAT-6/CFP-10 and the latency associated proteins Rv1733, Rv2029 and Rv2628 (Table 2). The role of IFN-γ during TB disease was evident as in response to all antigens, IFN-γ levels were highest, followed by Granzyme B (Figure 1). The same trend was observed at two weeks of treatment. Generally median cytokine levels increased from baseline to week 2 with only the increase in Rv1733-specific production of Granzyme B (P = 0. 013) being significant.

**Table 2:** **Positive cytokine responses per antigen before and after 2 weeks of TB treatment**

	No. of positive responders per antigen [n^1^/N^2^(%)]				
	SEB	E6/C10	Rv1733	Rv2029	Rv2628
**Granzyme B**					
Before	19/19 (100)	13/19 (68.4)	13/18 (72.2)	7/11 (63.6)	7/10 (70)
After 2wks	14/15 (93.3)	12/15 (80.0)	9/10 (90.0)	7/7 (100.0)	5/5 (100)
P value	0.4412	0.6974	0.3746	0.1193	0.5055
**IFN-γ**					
Before	18/19 (94.7)	16/19 (84.2)	15/18 (83.3)	8/11 (72.7)	8/10 (80.0)
After 2wks	12/15 (80.0)	13/15 (86.6)	10/10 (100)	7/7 (100)	5/5 (100)
P value	0.2994	1.0	0.5330	0.2451	0.5238
**IL-10**					
Before	18/19 (94.7)	14/19 (73.7)	12/18 (66.7)	7/11 (63.6)	5/10 (50.0)
After 2wks	12/15 (80)	9/15 (60.0)	7/10 (70.0)	4/7 (57.1)	5/5 (100)
P value	0.2994	0.4748	1.0	1.0	0.1009
**IL-17**					
Before	19/19 (100)	13/19 (68.4)	14/18 (77.8)	6/11 (54.5)	8/10 (80.0)
After 2wks	13/15 (86.6)	12/15 (80.0)	9/10 (90.0)	6/7 (85.7)	5/5 (100)
P value	0.1872	0.6974	0.6264	0.3156	0.5238
**sIL-2R-α**					
Before	18/19 (94.7)	14/20 (70.0)	12/18 (66.7)	7/11 (63.6)	8/10 (80.0)
After 2wks	11/15 (73.3)	12/15 (80.0)	9/10 (90.0)	6/7 (85.6)	5/5 (100)
P value	0.1458	0.7003	0.3642	0.5956	0.5238
**TNF-α**					
Before	16/19 (84.2)	13/19 (68.4)	12/18 (66.7)	7/11 (63.6)	7/10 (70.0)
After 2wks	11/15 (73.3)	9/10 (90.0)	9/10 (90.0)	5/7 (71.4)	5/5 (100)
P value	0.6722	0.3667	0.3642	1.0	0.5055

Positive cytokine responses per antigen at baseline and 2 weeks of anti-TB therapy. Freshly isolated PBMC from Sputum smear positive TB patients were stimulated for 6 days before initiation of therapy (baseline) with SEB (n = 19), ESAT-6/CFP-10 (n = 19), Rv1733 (n = 18), Rv2029 (n = 11) and Rv2628 (n = 10) and *after 2 weeks on anti-TB therapy* with SEB (n = 15), ESAT-6/CFP-10 (n = 15), Rv1733 (n = 10), Rv2029 (n = 7) and Rv2628 (n = 5). Culture supernatant was assessed by multiplex cytokine analysis. Percentage of positive cytokine (Granzyme B, IFN-γ, IL-10, IL-17, sIL2R-α and TNF-α) responses per antigenic stimulation were calculated. Positive responses were determined as follows; *Cytokine concentrations were divided by the negative control sample (medium only, un-stimulated) and values greater than or equal to 2 were considered positive*.

^1^Number of positive responses.

^2^Number of samples analyzed.

### Cytokine response to ESAT-6/CFP-10 fusion protein and latency associated Rv1733

**Figure 2:**
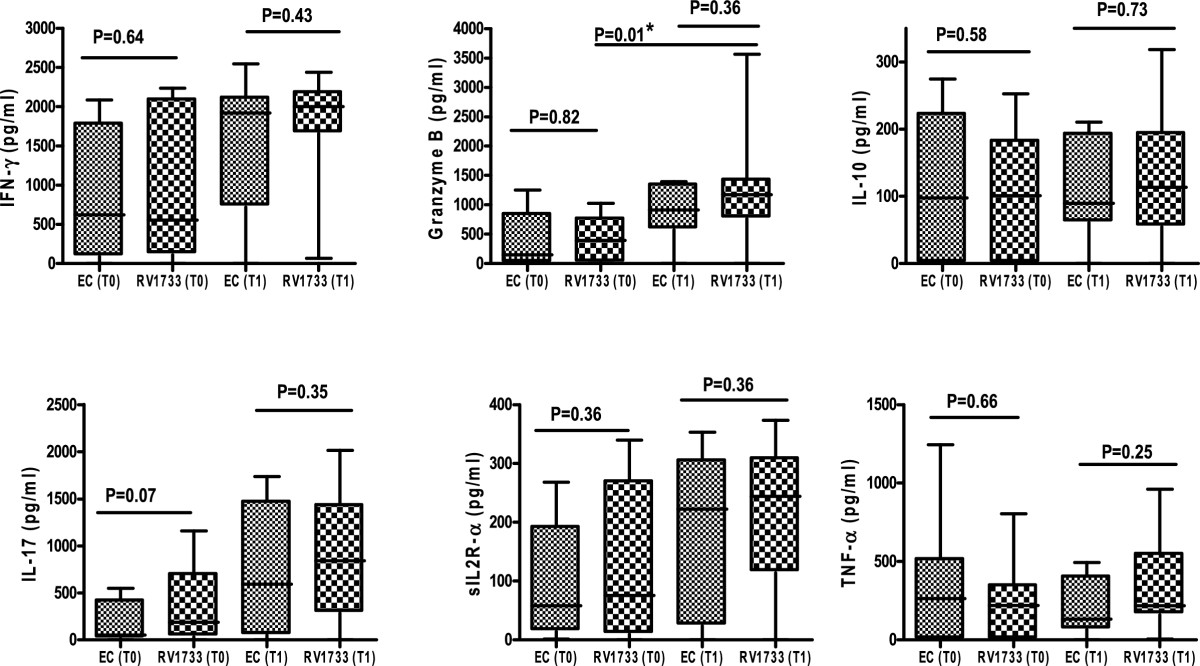
**ESAT-6/CFP-10 fusion protein and latency associated Rv1733 induce comparable levels of the 6 cytokines.** Cytokine levels induced by ESAT-6/CFP-10 (EC) fusion protein and Latency associated Rv1733 at baseline (T0) and 2 weeks (T1) of treatment were compared using the Wilcoxon matched pairs test in patients who had data for both time points available (n = 9). Cytokine levels were obtained after subtracting values in un-stimulated wells from stimulated wells and negative values were converted to zero. The box plots show the 25th, 50th, and 75th percentiles, and the whiskers represent the minimum and maximum levels of cytokine (pg/ml) induced by each stimulus. For each cytokine, the comparison was done between the amount secreted by ESAT-6/CFP-10 vrs Rv1733 at baseline (T0) and that between ESAT-6/CFP-10 vrs Rv1733 at week two (T1). P values of <0.05 were considered significant.

To determine whether differences exist in the cytokine response profile to antigens associated with replicating (ESAT-6/CFP-10) and latent (Rv1733) bacilli, the Wilcoxon matched pairs test was used to compare the levels of each cytokine released in response to the two antigens at baseline (T0) and 2 weeks (T1) of treatment. This analysis included only patients (n = 9) with complete data for all cytokines at both time points. There was no difference (P > 0.05) in levels of the 6 cytokines induced by ESAT-6/CFP-10 and the latency associated Rv1733 at baseline (T0) and 2 weeks of treatment (T1). Median concentration (pg/ml) of all cytokines (except TNF-α) in response to ESAT-6/CFP-10 were higher at TI compared to T0 and the same trend was observed with responses to Rv1733. However, only the median increase of Rv1733-specific Granzyme B response was of statistical significance (P = 0. 01) (Figure 2).

### Cytokine response to antigenic stimulation in individual patients

**Figure 3:**
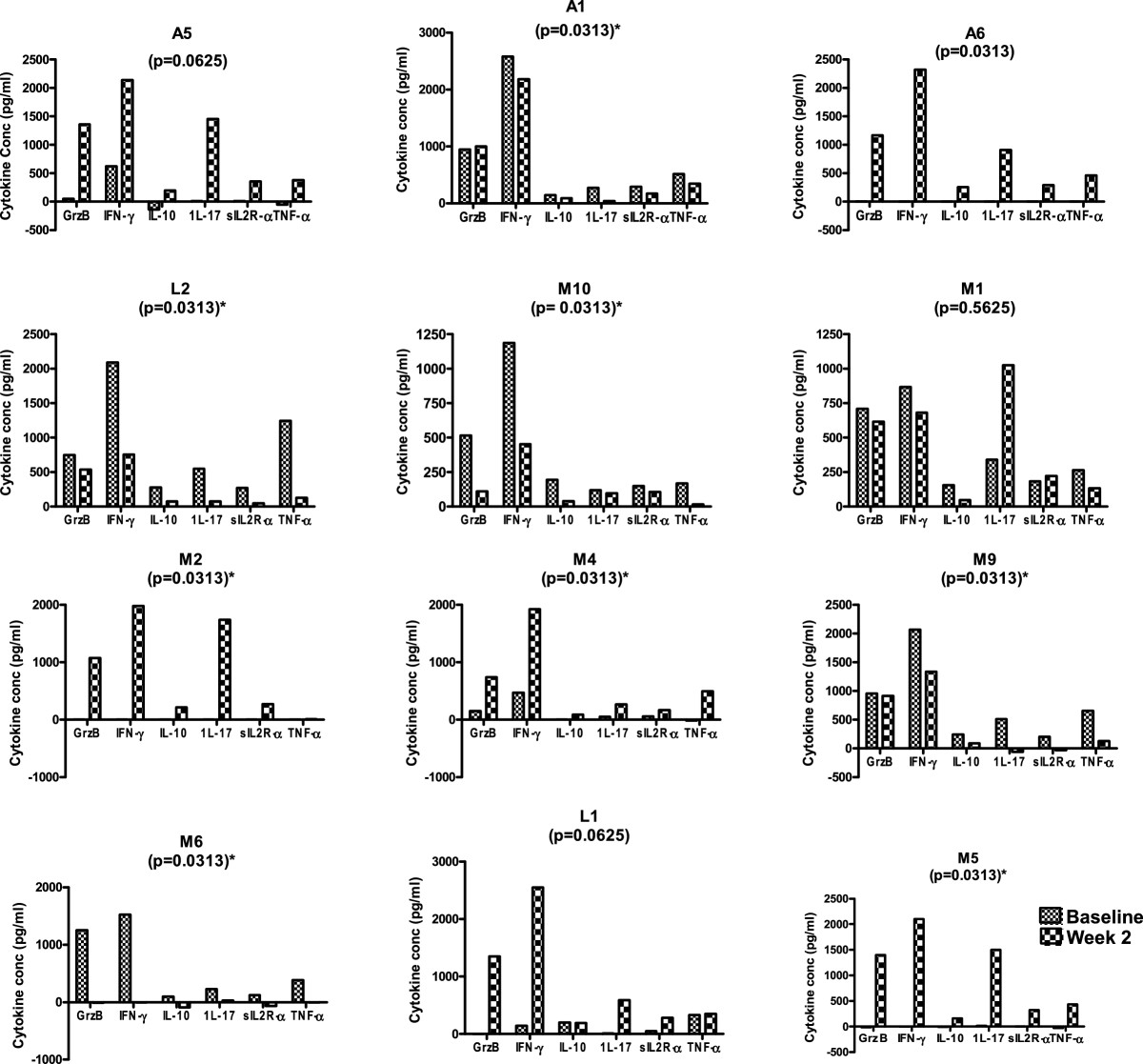
**Changes in cytokine levels after 2 weeks of TB treatment follows three patterns.** Cytokine levels at baseline and after two weeks of treatment in response to each antigen were determined for each patient. Cytokine levels were calculated by subtracting the background (value of un-stimulated control) from measured values for each cytokine. Data was analyzed using Wilcoxon signed rank test and P values <0.05 were considered significant. Shown are the cytokine profiles of patients (n = 12) who had both time points available for response to ESAT-6/CFP-10. In patients A1, L2, M10, M9 there is a significant *decrease* in all cytokine levels at week two, while in patients A6, M4, M2, M6, M5 there is a significant *increase* in all cytokine levels. The increase and decrease in cytokine levels in A5 and L1 respectively, are not significant while M1 shows fluctuating levels.

To determine if the observed improvement in cellular responses was a reflection of individual cytokine responses and not a group effect, analysis of cytokine responses to ESAT-6/CFP-10 at baseline and 2 weeks of treatment was done for individual patients (n = 12) who had both data available. A wide inter-individual variation was observed in cytokine response profile. As such, cytokine responses to ESAT-6/CFP-10 were categorized based on the three distinct response patterns observed from baseline to week 2 as; (a) Increased median cytokine concentration, (b) decreased median cytokine concentration and (c) Fluctuations in median concentration for all 6 cytokines (Figure 3). To understand this variation in cytokine response pattern, a Uni-variate analysis was done for “age” and “sputum smear result at diagnosis” with cytokine response to antigenic stimulation (increased/decreased/fluctuating) as outcome variables. However, no association could be established.

### Dynamics of MTB-specific CD4 and CD8 T cell responses during the first two weeks of treatment

**Figure 4:**
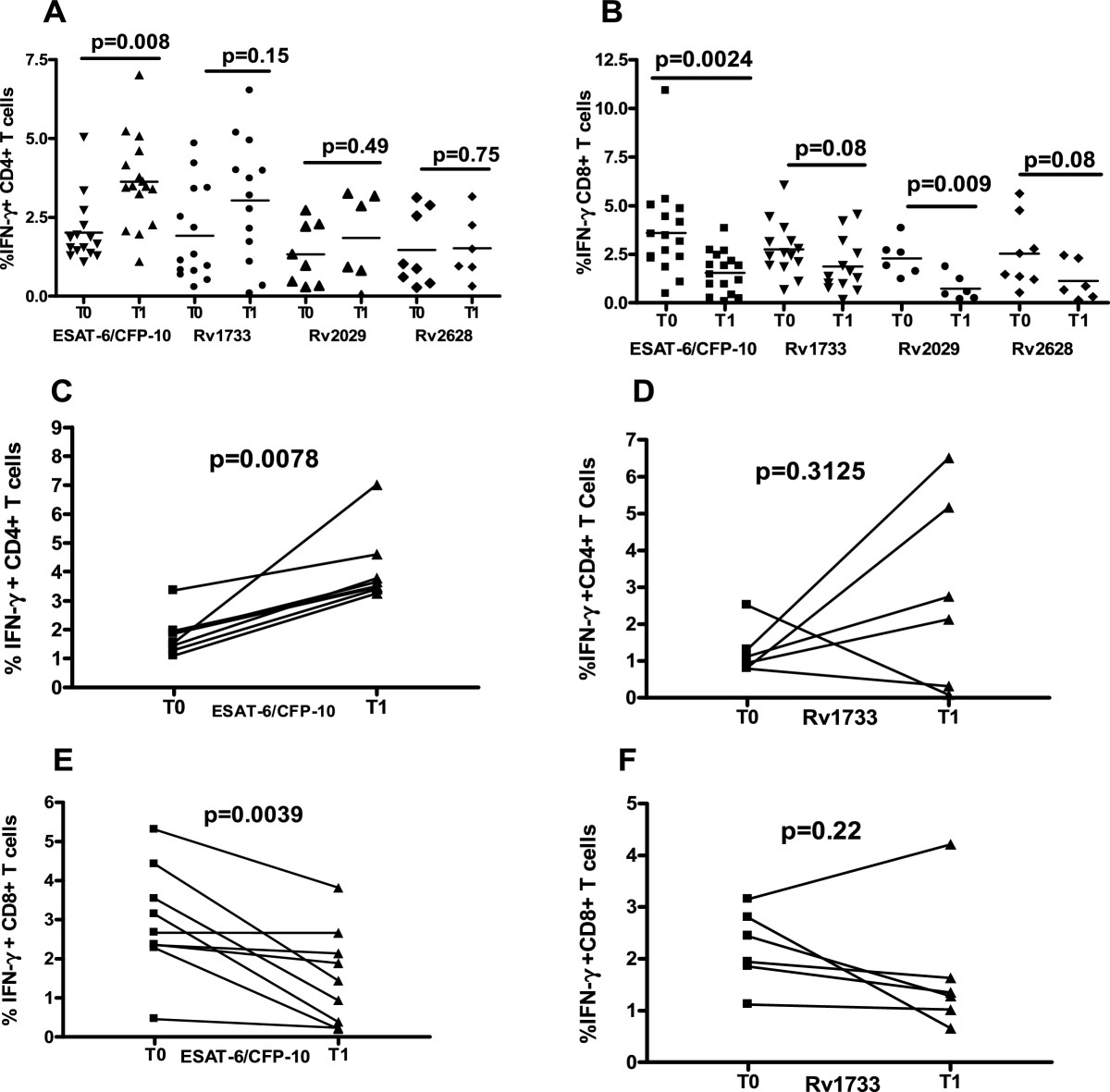
**Median frequencies of IFN-γ + CD4 and CD8 T cells in PBMC of TB patients before and after 2 weeks of anti-TB therapy.** Freshly isolated PBMC from newly diagnosed sputum smear positive and HIV-negative TB patients (n = 19) were stimulated for 6 days with ESAT-6/CFP-10, Rv1733, Rv2029, Rv2628, SEB and the un-stimulated control (Medium). IFN-γ + CD4+ and IFN-γ + CD8+ T cells were assessed by flow cytometry by subtracting the percentage in un-stimulated cultures from stimulated ones. A threshold of 0.2% IFN-γ + CD4/ CD8 T cells defined positive T-cell responses against antigens. The Mann–Whitney U test (P < 0.05) was used to compare frequency of IFN-γ + CD4 **(A)** and CD8 **(B)** T cells at baseline and week two in response to ESAT-6/CFP-10 (n = 11), Rv1733 (n = 10), Rv2029 (n = 6), Rv2628 (n = 6). Also shown is the frequencies IFN-γ + CD4 **(C&D)** and CD8 **(E&F)** T cell at baseline (T0) and at 2 weeks (T1) into treatment for individual participants with data available for response to ESAT-6/CFP-10 (n=8) and Rv1733 (n=6). P values computed with the Wilcoxon signed rank test (P<0.05).

To determine the functional T cell phenotypes contributing to the cytokine secretion during the first two weeks of treatment, we assessed the IFN-γ + CD4+ and CD8+ T cells at baseline and at two weeks of treatment in the same patients. After two weeks of treatment, the median frequency of antigen specific IFN-γ + CD4 T cell responses increased compared to baseline values; however, only the increase in the ESAT-6/CFP-10-specific response was significant (P = 0. 0008). In contrast, the median frequency of antigen specific IFN-γ + CD8 T cell responses declined during week two, with the decline in ESAT-6/CFP-10 -specific (P = 0.0024) and Rv2029- specific CD8 T cell response (P < 0.009) being significant (Figure 4A and B). The Wilcoxon matched pairs test analysis was done on subjects for whom T cell responses at both time points in response to ESAT-6/CFP-10 and Rv1733 (the strongest inducer of T cell responses among the 3 DosR proteins) were positive. Again the frequency of IFN-γ + CD4 T cells in response to ESAT-6/CFP-10 was significantly increased (P = 0. 0078) at week two, while the frequency of IFN-γ + CD8 T cells was significantly decreased (P = 0. 0039) (Figure 4C). There were no significant changes in response to Rv1733 (Figure 4D).

## Discussion

Cytokine secretion profile in response to mycobacterial antigens has been extensively studied in identifying immunological differences between active TB and latent infection or in TB patients before and after treatment. The first two weeks of TB treatment are known for the rapid reduction of metabolically active mycobacteria, yet studies on changes in the host immunological response during this period are scanty. Such changes could be exploited in the design of putative biomarkers for early treatment response. Determining the cytokine secretion profile of Mtb-specific antigens in different settings would also expand our knowledge of the performance of potential immunogenic *M. tuberculosis* antigens which could be worthy candidates for designing new diagnostics or vaccines [17]. In this study, the four tested MTB antigens were immunogenic in our cohort as evidenced by the highly significant differences (P < 0.01 to P < 0.0001) seen between stimulated and un-stimulated cultures.

Consistent with other studies, [13, 18, 19] cytokine levels in response to ESAT-6/CFP-10 fusion protein stimulation were generally high. ESAT-6 is a virulence factor secreted by actively replicating bacteria early during infection, so high responses in TB patients at the early stage of disease at are not unusual. However, cytokines were secreted in response to the latency associated antigens as well. The recognition of the latency-associated antigens by PBMC from active TB patients could reflect the fact that most TB patients undergo a latent infection prior to TB disease [20] or indicate the simultaneous involvement of latent and metabolically active bacteria in active disease. Thus an individual could harbour actively replicating bacteria and succumb to TB, while having latent foci that will lead to recognition of latency related antigens. The immunodominance of Rv1733 across populations was affirmed as in agreement with other studies, [13, 18], Rv1733 was the most recognized (of the 3 DosR antigens used) in our cohort in terms of positive cytokine responders and frequency of IFN-γ + T cell response.

The cytokine profile in response to all antigenic stimulation consisted of high levels of IFN-γ followed by grzB and TNF-α and lower levels of IL-17, sIL2Rα and IL-10 (Figure 1). It would be expected that ESAT-6/CFP-10 being virulent factors would induce both pro and anti inflammatory cytokines whilst the dormancy -related Rv1733, may only induce pro inflammatory responses and relatively little anti-inflammatory response. This actually confirms that the immune responses during the active stage of the disease (TB patients) is characterized by both pro and anti-inflammatory cytokines irrespective of the nature of the antigen. While the high IFN-γ levels were expected due to its critical role in the control of Mtb infection, [21–23] the higher levels of Granzyme B in this study in response to all antigens was an interesting finding. In a previous study by Toosii et al. [24] IFN-y and grzB were the only Mtb effector molecules that were inducible in PBMC from Mtb-sensitized subjects. In Mtb infections, the combined action of perforin and the antibacterial agent granulysin, both of which are expressed in the granules of CTLs and NK cells, influences the outcome of infection [25]. Studies using mouse models indicate that the majority of Mtb-specific CD8 + T cells are limited to either cytotoxicity or the secretion of IFN-γ, [26]. High levels of grzB could indicate that the early stage of disease control is characterized by lysis of infected cells to quell the spread of infection and re-enforces the role of CD8+ T cells in the control of human tuberculosis infection. This was evident as we saw a higher frequency of CD8+ T cells than CD4+ at baseline and a decline in CD8+ T cells at week two and an increase in IFN-γ + CD4 T cells. Marginal increases in cytokine levels from baseline to week two as well as a significant increase in the frequencies of CD4+ T cells was observed, suggestive of an improvement in cellular response with therapy. However, only the increase in median Rv1733- specific Granzyme B secretion was of statistical significance. Although effect of time point and antigen on cytokine secretion could be observed most of the trends were non-significant. It could be because the sample size was too small to show any differences or cytokines assessed remain relatively unchanged or stable from baseline to week two of treatment. Improvement in cellular response with therapy has been reported in previous studies [27–30].

Two reasons have been adduced for this cellular improvement, firstly an increase in the number of peripheral CD4+ T cells that produce IFN-γ, owing to the fact that CD4+ T cells responsive to a vast array of Mtb epitopes are sequestered or compartmentalized at the site of the disease, and appear in the peripheral blood after effective chemotherapy, which reverses the state of anergy seen in these patients [27, 30]. This is actually corroborated by our study as the median frequency of IFN-γ + CD4 T cells increased during week 2 from baseline levels. Secondly a shift in cytokine production by PBMC from cytokines that down regulates the activation of Th1 cells and their cytokines such as IL-10. The levels of these regulatory cytokines are high in active TB patients and decrease upon treatment with anti-TB drugs [31, 32].

In-spite of this general improvement in cytokine responses, inter-individual variation was observed with 3 distinct patterns of Increased, decreased or fluctuating levels of all cytokines. We could not detect an association between “age” and “sputum smear result at diagnosis” with cytokine response to antigenic stimulation. The variation could be best explained by host intrinsic factors beyond the scope of this study.

This study had some limitations, including the small sample size (n = 20), and limited number of analytes tested for (6 cytokines). Also, we could not take sputum samples at week 2 which would have permitted us to directly compare the changes in the cytokine profile to smear status (bacterial load) at week 2 and perhaps would have helped to better explain the individual variation in response. Also, epidemiological factors were not considered.

## Conclusion

In conclusion, effective chemotherapy improved cellular responses of TB patients to Mtb stage specific antigens, as early as two weeks of anti-TB therapy, characterized by a general increase in secretion of various cytokines. However, using cytokine levels to predict early treatment response to anti-TB treatment will be difficult due to the wide inter-individual variation observed. While the amount of cytokines produced may not be an indication of its biological activities, the high quantities of Granzyme B warrants further investigation of its role in the control of TB infection and the possible utility as a marker for early TB treatment response.

## Authors’ original submitted files for images

Below are the links to the authors’ original submitted files for images. Authors’ original file for figure 1 Authors’ original file for figure 2 Authors’ original file for figure 3 Authors’ original file for figure 4
