# Supplementary figures and images for: Cytokine response to selected MTB antigens in Ghanaian TB patients, before and at 2 weeks of anti-TB therapy is characterized by high expression of IFN-γ and Granzyme B and inter- individual variation
Source: BMC Infect Dis. 2014 Sep 10;14:495. doi: 10.1186/1471-2334-14-495 (PMC4180837; doi:10.1186/1471-2334-14-495)

## GROWTH MEDIUM

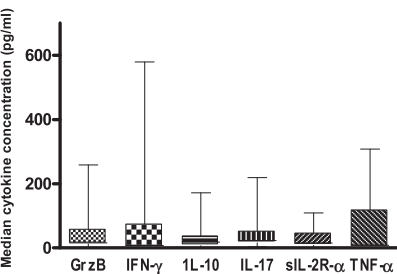

## SEB

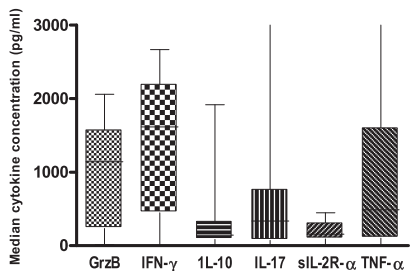

## ESAT-6/CFP-10

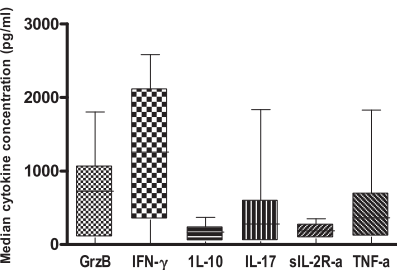

## Rv1733

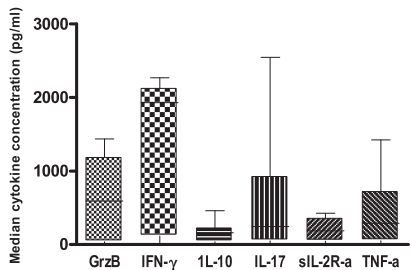

## Rv2029

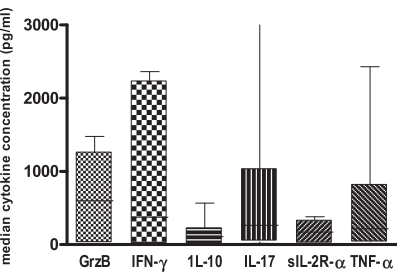

## Rv2628

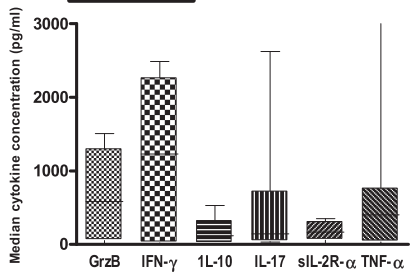

Supplement: Supplementary file 1 — Authors’ original file for figure 1 [file 12879_2014_3828_MOESM1_ESM.pdf]

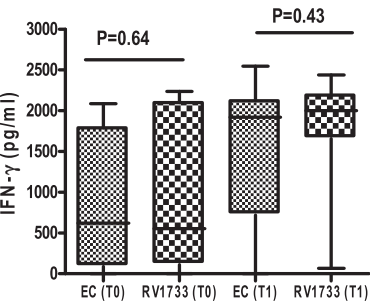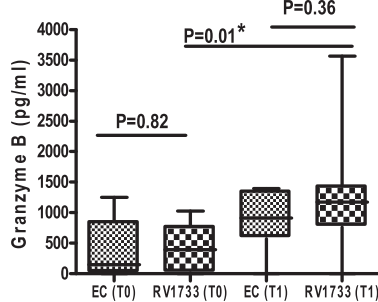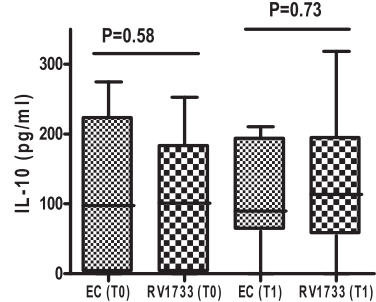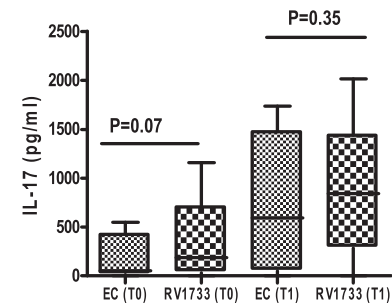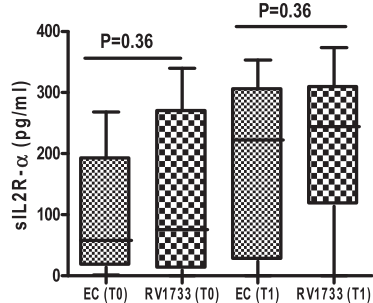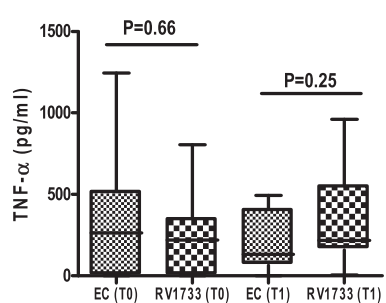

Supplement: Supplementary file 2 — Authors’ original file for figure 2 [file 12879_2014_3828_MOESM2_ESM.pdf]

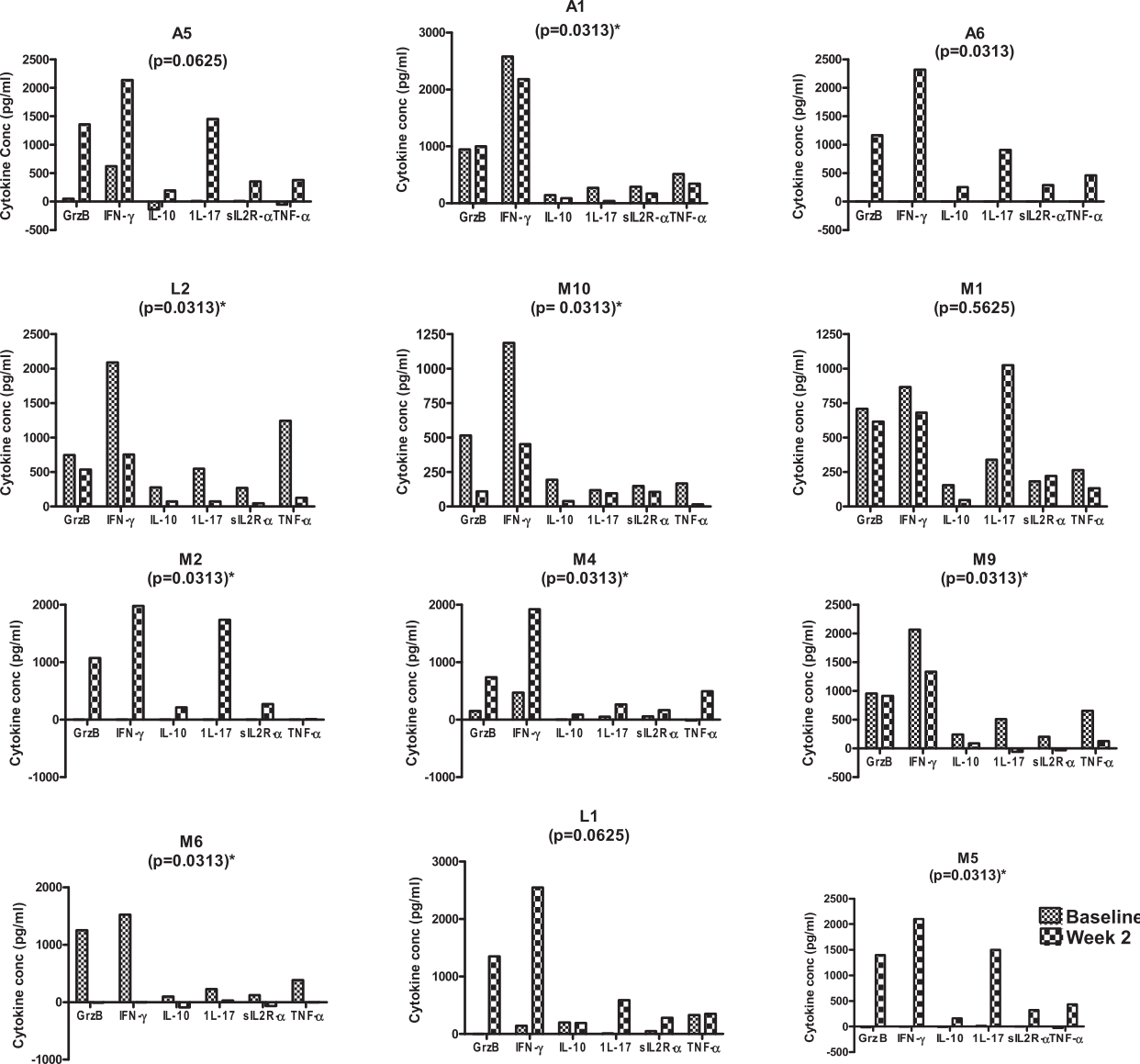

Supplement: Supplementary file 3 — Authors’ original file for figure 3 [file 12879_2014_3828_MOESM3_ESM.pdf]

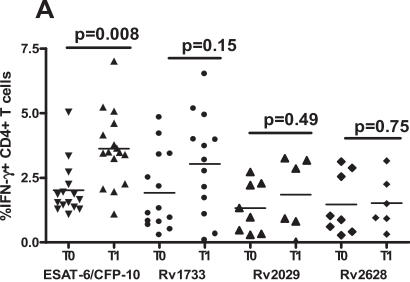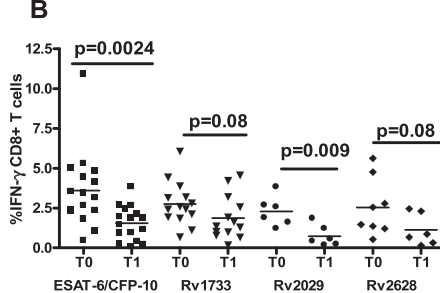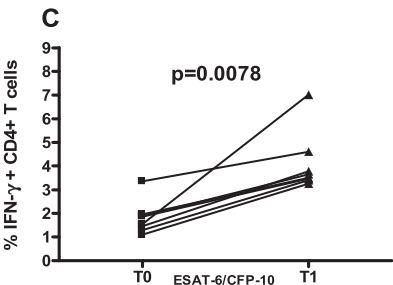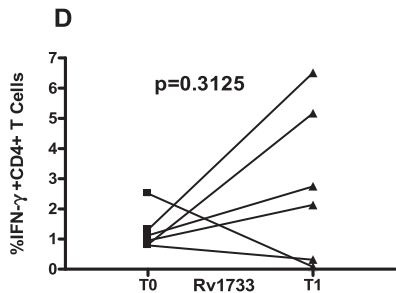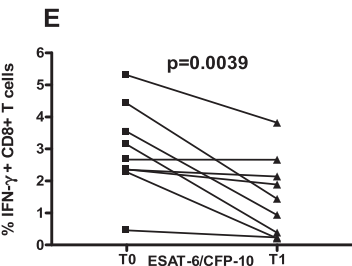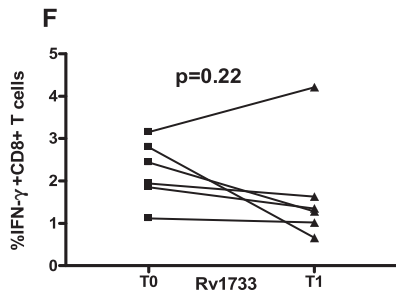

Supplement: Supplementary file 4 — Authors’ original file for figure 4 [file 12879_2014_3828_MOESM4_ESM.pdf]
